# Pleomorphic xanthoastrocytomas of adults: MRI features, molecular markers, and clinical outcomes

**DOI:** 10.1038/s41598-018-32273-w

**Published:** 2018-09-24

**Authors:** Jing Yan, Jingliang Cheng, Furong Liu, Xianzhi Liu

**Affiliations:** 1 Department of MRI, The First Affiliated Hospital of Zhengzhou University, Zhengzhou, 450000 China; 2 Department of Medical Records Management, The First Affiliated Hospital of Zhengzhou University, Zhengzhou, 450000 China; 3 Department of Neurosurgery, The First Affiliated Hospital of Zhengzhou University, Zhengzhou, 450000 China

## Abstract

Fifty adult patients with pathologically-confirmed pleomorphic xanthoastrocytomas (PXAs) were retrospectively analyzed. Adult PXAs appeared as a single lesion in 47 patients and multiple lesions in 3 patients. Temporal lobe was the most common location (17/50). Twenty-two cases were superficial with obvious meningeal contact and 9 were closely adjacent to lateral ventricles. Three imaging patterns were differentiated, including a predominantly solid mass with or without cystic changes (n = 33), a predominantly cystic mass with an obvious mural nodule (n = 14), and a predominantly cystic mass with an uneven wall thickness (n = 3). The mean tumoral apparent diffusion coefficient (ADC) was 0.83 ± 0.17 × 10^−3^ mm^2^/s, and the mean ADC ratio was 1.02 ± 0.22. The V-raf murine sarcoma viral oncogenes homolog B1 (BRAF)^V600E^ mutation was found in 12 of 29 patients. In 36 patients with isocitrate dehydrogenases 1 and 2 (IDH1/2) data, only one had IDH1 mutation and no patient had IDH2 mutation. Anaplastic features were common (24/50) and significantly associated with high rates of recurrence or progression (*P* < 0.001). In conclusion, this study expands our knowledge on the MRI features, molecular markers, and clinical outcomes of adult PXAs, to some extent different from pediatric PXAs.

## Introduction

Pleomorphic xanthoastrocytomas (PXAs) are rare, slow-growing central nervous system (CNS) neoplasms that account for less than 1% of astrocytomas. They predominantly occur in children and young adults with a mean age of 26 years^1,2^. Moreover, nearly 2/3 of cases occur in patients younger than 25 years old^2^. Several previous studies have reported the imaging features, histopathology, and treatment of PXAs^3–9^, however, the reports dedicated solely to adult PXAs are rare. Histologically, a PXA has a pleomorphic appearance with spindle cells being admixed with mono- and multi-nucleated giant cells. The presence of cells with xanthomatous changes due to the intracellular accumulation of lipid droplets is common^1,2^. The histopathological descriptor “pleomorphic” that is applied in radiology, molecular biology, and prognosis of PXAs results in myriad presentations of PXAs, potentially complicating the diagnosis and therapeutic interventions of PXAs.

Due to the difficult diagnosis and therapeutic challenge of PXAs, in this study, we focused on a cohort of 50 adult patients diagnosed with PXAs to search for magnetic resonance imaging (MRI) features, molecular markers, and clinical outcomes of adult PXAs.

## Results

### Patient characteristics

Of these 50 patients, 31 were males and 19 were females. The median age at diagnosis was 36 years, ranged from 18 to 67 years. Headaches were the most frequent symptoms at diagnosis and were seen in 24 patients (48%), followed by seizures in 15 patients (30%), focal neurological deficits in 13 patients (26%), and visual disturbances in 11 patients (22%). The duration of symptoms ranged from 4 days to 20 years.

All 50 patients underwent surgical resection of the tumor. Gross total resection was achieved in 37 cases, subtotal resection was achieved in 10 cases, and partial resection was achieved in 3 cases (Table 1).

**Table 1:** Individual patient information on demographics, histology, gene, and outcomes.

Variable	Number (n = 50)
**Median age (years)**	36 (18–67)
**Gender**	
Male	31 (62%)
Female	19 (38%)
**Symptoms**	
Seizures	15 (30%)
Headaches	24 (48%)
Focal neurological deficits	13 (26%)
Visual disturbances	11 (22%)
**Extent of surgery**	
Gross total resection	37 (74%)
Subtotal resection	10 (20%)
Partial resection	3 (6%)
**Histopathology**	
Grade II PXA	26 (52%)
Grade III anaplastic PXA	24 (48%)
**BRAF** ^**V600E**^ **status**	
Mutated	12 (41%)
Non-V600E mutated	17 (59%)
N/A	21
**IDH1 status**	
Mutated	1 (3%)
Non-mutated	35 (97%)
N/A	14
**IDH2 status**	
Mutated	0
Non-mutated	36 (100%)
N/A	14
**Clinical outcomes**	
Complete remission	16 (53%)
Recurrence or progression	14 (47%)
N/A	20

N/A = not available.

### Variables observed on MRI

**Figure 1:**
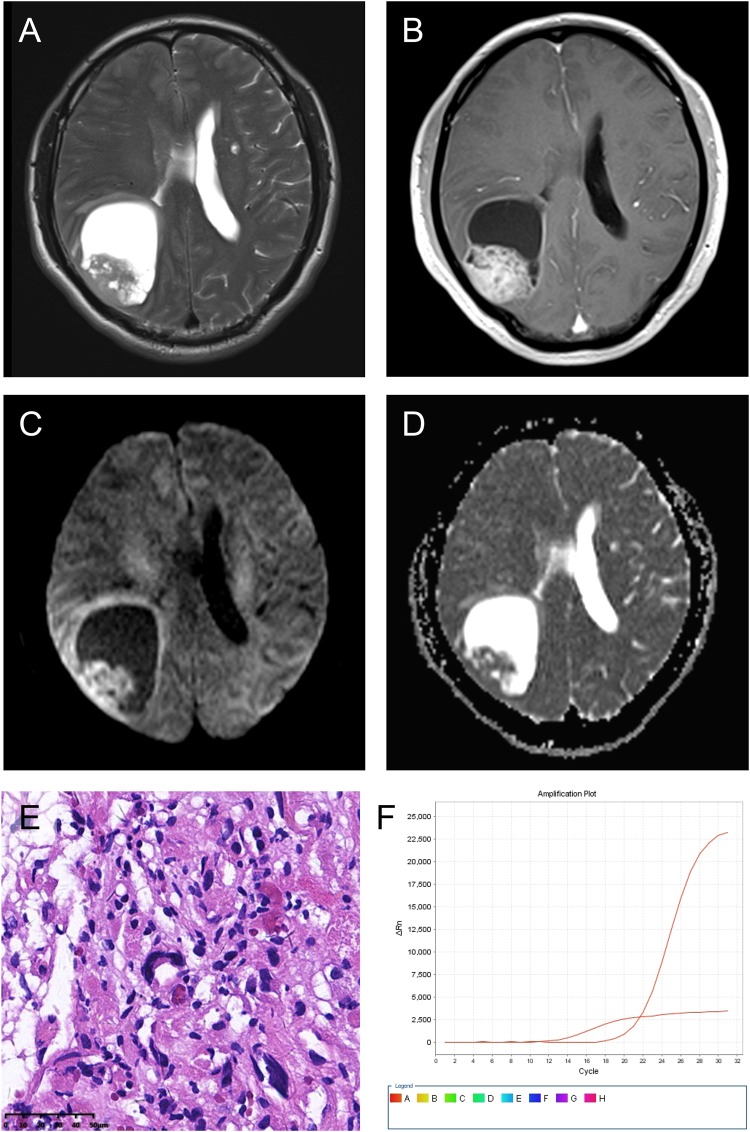
A right parietal-occipital lobe Grade III anaplastic PXA in a 46-year-old male with a predominantly cystic mass and a mural nodule close to the meningeal side. (**A**) Axial T2WI showing the iso-intense mural nodule and the homogeneously hyper-intense cystic component. (**B**) Contrast-enhanced axial T1WI showing marked enhancement of the mural nodule and cystic wall. (**C**) DWI showing restricted diffusion of the mural nodule. (**D**) The mean ADC of the mural nodule was 0.87 × 10^−3^ mm^2^/s, and the mean ADC ratio was 1.08. (**E**) Photomicrography of the tumor showing marked cellular pleomorphism, with spindle cells, multi-nucleated giant cells with bizarre nuclei, and lipid-filled xanthomatous cells. Significant mitotic activity was present (H&E, ×400). (**F**) Molecular detection showing the BRAF^V600E^ mutation.

**Figure 2:**
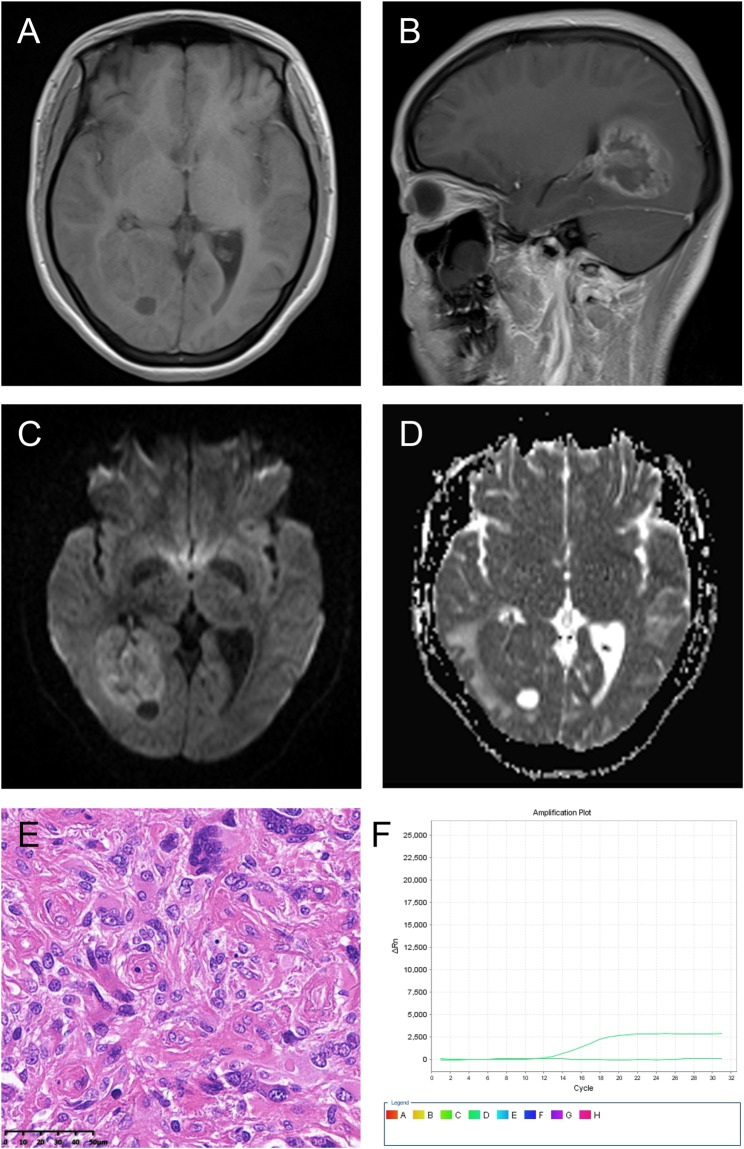
A right temporal-occipital lobe Grade III anaplastic PXA in a 23-year-old female with a predominantly solid mass and cystic changes. (**A**) Axial T1WI showing the iso-intense of the solid component. The lesion was closely associated with the lateral ventricle and even invasion of the temporal horn of the lateral ventricle. (**B**) Contrast-enhanced sagittal T1WI showing marked enhancement of the solid component. (**C**) DWI showing restricted diffusion of the solid component. (**D**) The mean ADC of the mural nodule was 0.75 × 10^−3^ mm^2^/s, and the mean ADC ratio was 0.92. (**E**) Photomicrography of the tumor showing marked cellular pleomorphism with spindle cells, multi-nucleated giant cells with bizarre nuclei, and lipid-filled xanthomatous cells. Significant mitotic activity was present (H&E, ×400). (**F**) Molecular detection showing the BRAFV^600E^ mutation not detected.

**Figure 3:**
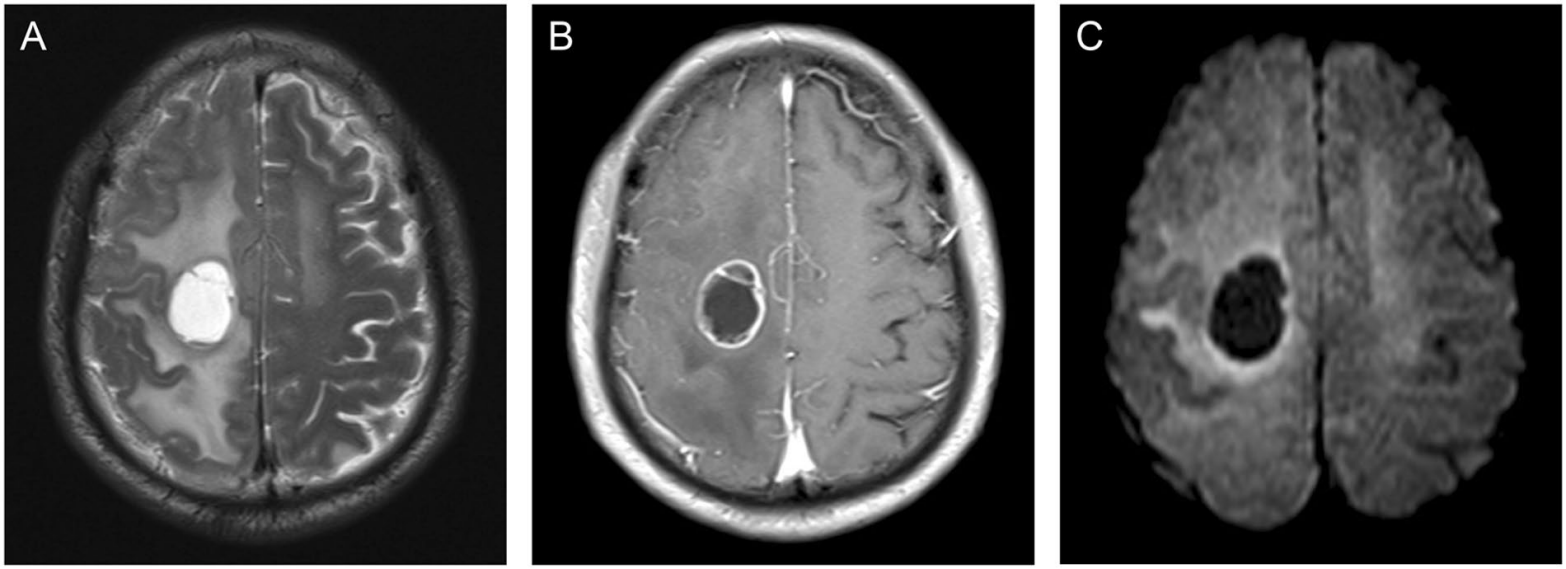
A right frontal lobe Grade III anaplastic PXA in a 51-year-old male with a predominantly cystic mass without an obvious mural nodule. (**A**) Axial T2WI showing the iso-intense cystic wall and homogeneous hyper-intense appearance of the cyst-like area. Obvious vasogenic edema surrounded the mass. (**B**) Contrast-enhanced axial T1WI showing the cystic wall with intense enhancement and the cyst-like area without enhancement. (**C**) DWI showing no evidence of restricted diffusion of the tumor.

**Figure 4:**
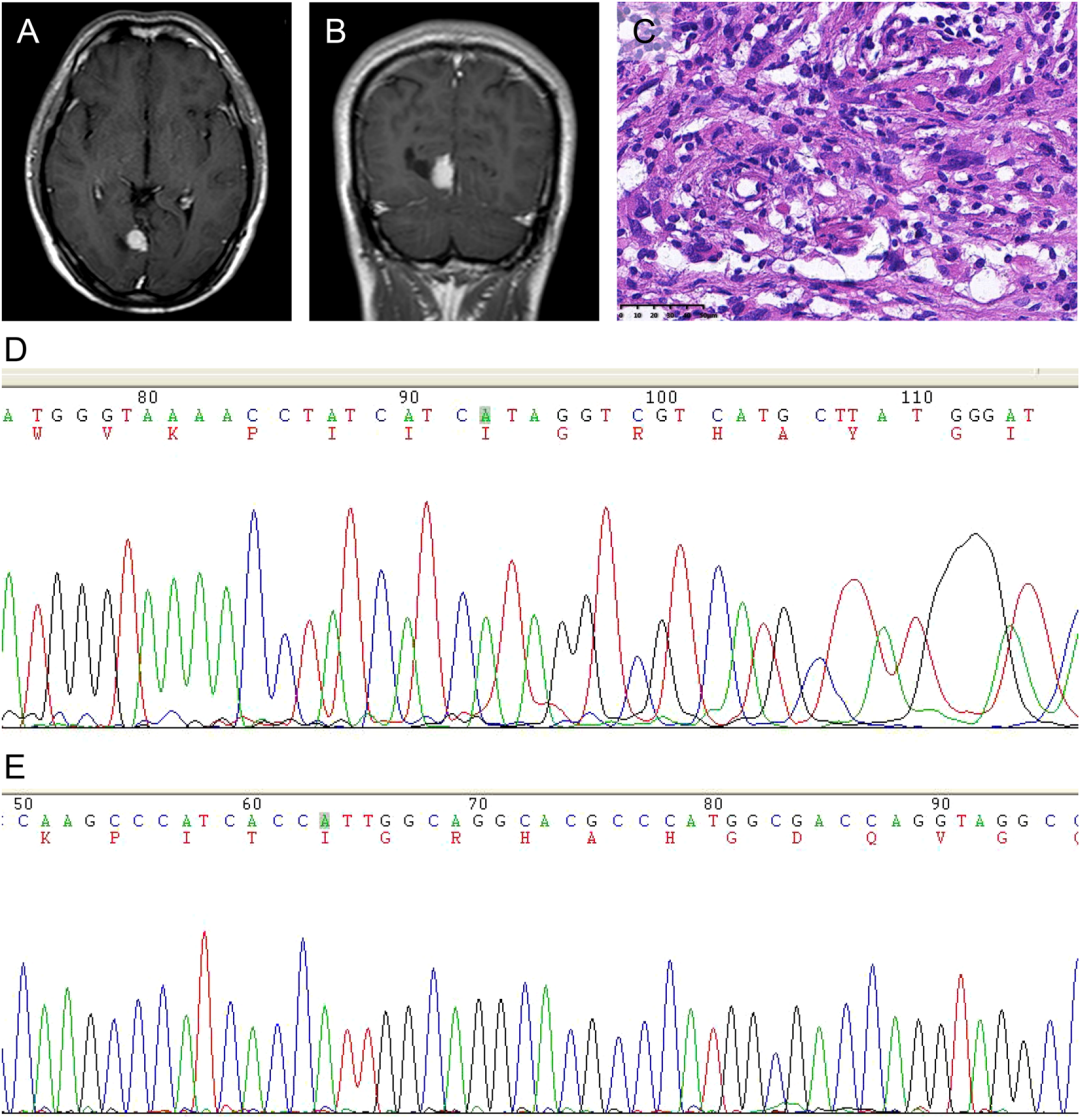
A right occipital lobe Grade II PXA in a 25-year-old male with a predominantly solid mass without cystic changes. (**A**,**B**) Contrast-enhanced axial and coronal T1WI showing marked enhancement of the solid component. (**C**) Photomicrography of the tumor showing marked cellular pleomorphism with spindle cells, multinucleated giant cells containing bizarre nuclei, and lipid-filled xanthomatous cells. Mitotic activity and necrosis were not present (H&E, ×400). (**D**,**E**) Molecular detection showing the IDH1/2 mutations not detected.

**Figure 5:**
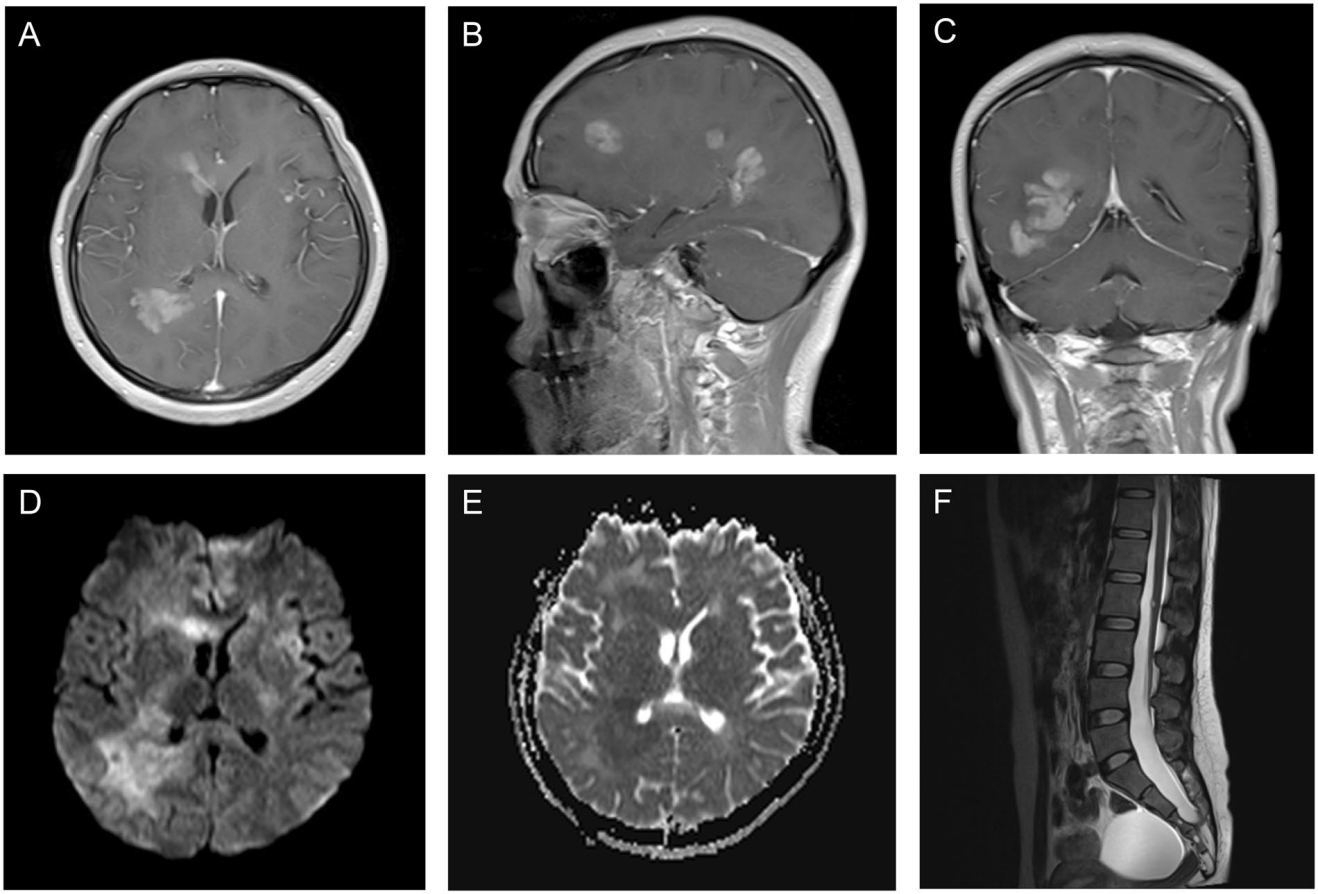
A Grade II PXA involving the right frontal lobe, right temporal-parietal lobe, corpus callosum, and left insula in a 25-year-old female. (**A**–**C**) Contrast-enhanced axial, sagittal, and coronal T1WI showing marked enhancement of multiple lesions in the brain. (**D**) DWI showing restricted diffusion of multiple lesions. (**E**) The mean ADC of the right temporal-parietal lobe lesion was 0.80 × 10^−3^ mm^2^/s, and the mean ADC ratio was 1.01. (**F**) Sagittal T2WI depicting a hyper-intense spinal intramedullary lesion at the L2 level.

As shown in Table 2, the maximum diameter of the tumor was 13–80 mm. A single lesion (Figs 1–4) was found in 47 cases (94%). Tumors were predominantly located in the supratentorial cerebral hemisphere with 43 patients (91%), and temporal lobe was the most common tumor site with 17 patients (36%). Multiple lesions were found in 3 cases, and 1 case was concomitant with a spinal intramedullary lesion at the L2 level (Fig. 5). Twenty-two cases (44%) were superficial in the cerebral hemisphere and attached to the meninges (Fig. 1). The locations of 9 cases (18%) with supratentorial tumors were adjacent to and even invasion of the lateral ventricles (Figs 2 and 5). PXAs were also located in the lateral ventricles (n = 2), dorsal midbrain (n = 1), and thalamus (n = 1). Hemorrhage was observed in 9 cases (18%). Peritumoral edema was observed in 41 cases, 30 of which had moderate to severe (≥10 mm in diameter) peritumoral edema.

**Table 2:** Variables observed on MRI.

Variable	Number (n = 50)
**Median size (mm)**	44 (13–80)
**Number**	
Single lesion	47 (94%)
Multiple lesions	3 (6%)
**Location**	
** Single lesion (n = 47)**	
Frontal lobe	12 (26%)
Temporal lobe	17 (36%)
Parietal lobe	4 (9%)
Occipital lobe	1 (2%)
Frontal-temporal lobe	3 (6%)
Temporal-parietal lobe	1 (2%)
Temporal-occipital lobe	4 (9%)
Parietal-occipital lobe	1 (2%)
Lateral ventricle	2 (4%)
Dorsal midbrain	1 (2%)
Thalamus	1 (2%)
** Multiple lesions (n = 3)**	
Frontal lobe, temporal-parietal lobe, insular lobe and corpus callosum	1
Parietal lobe, temporal lobe	1
Frontal lobe, occipital lobe and lateral ventricle	1
**Superficial and attached to the meninges**	22 (44%)
**Adjacent to and invasion of the lateral ventricles**	9 (18%)
**Hemorrhage**	9 (18%)
**Degree of peritumoral edema**	
No	9 (18%)
Mild (<10 mm diameter)	11 (22%)
Moderate to severe (≥10 mm diameter)	30 (60%)
**Imaging pattern**	
Predominantly solid	33 (66%)
Predominantly cystic with a mural nodule	14 (28%)
Predominantly cystic without a mural nodule	3 (6%)
**Enhancement pattern in the solid component (n = 47)**	
Mild	1 (2%)
Marked	46 (98%)
**Enhancement pattern in the cystic wall (n = 17)**	
No	7 (41%)
Mild	2 (12%)
Marked	8 (47%)
**DWI parameters**	
Mean ADC (×10^−3^ mm^2^/s)	0.83 ± 0.17 (0.58–1.42)
Mean ADC ratio	1.02 ± 0.22 (0.68–1.66)

Three imaging patterns were differentiated: (1) a predominantly solid mass with or without cystic changes in 33 cases (Figs 2, 4 and 5); (2) a predominantly cystic mass with a mural nodule in 14 cases, with the mural nodule close to the meningeal side in 8 cases (Fig. 1); and (3) a predominantly cystic mass with an uneven wall thickness, without an obvious mural nodule in 3 cases (Fig. 3). In all 50 cases, the solid component and mural nodule appeared slightly hypo- or iso-intense on T1-weighted spin-echo imaging (T1WI), iso- or slightly hyper-intense on T2-weighted spin-echo imaging (T2WI), and iso- or slightly hyper-intense on T2-fluid-attenuated inversion recovery (FLAIR) sequence. The cystic component appeared homogeneously hypo-intense on T1WI, hyper-intense on T2WI, and slightly hypo-intense on T2 FLAIR. Marked contrast enhancement in the solid component and mural nodule was observed in 46 cases, and marked contrast enhancement in the cystic wall was observed in 8 cases.

The diffusion-weighed imaging (DWI) showed restricted diffusion in 11 cases (Figs 1C, 2C and 5D) and no evidence of restricted diffusion in 15 cases (Fig. 3C). Tumoral apparent diffusion coefficient (ADC) values were not calculated in 3 cystic cases without obvious mural nodules because there was not enough solid area to place the regions of interests (ROIs) without partial volume effects in the necrotic region. Therefore, tumoral ADC values were measured in only 23 patients. The mean tumoral ADC was 0.83 ± 0.17 × 10^−3^ mm^2^/s (min-max: 0.58–1.42). The mean ADC ratio was 1.02 ± 0.22 (min-max: 0.68–1.66).

### Histopathological and molecular findings

According to the World Health Organization (WHO) classification, 26 (52%) and 24 (48%) cases were histopathologically diagnosed with Grade II PXA and Grade III anaplastic PXA, respectively. Grossly, some tumors were superficial in the cerebral hemisphere and attached to the meninges, and some were accompanied by a large cystic component containing mural nodules. Microscopically, the WHO Grade II PXAs had a pleomorphic appearance with spindle cells being admixed with mono- and multi-nucleated giant cells. The presence of cells with xanthomatous changes was due to intracellular accumulation of lipid droplets. Mitotic activity was rare, and necrosis was usually absent (Fig. 4C). Tumors with significant mitotic activity (i.e., five or more mitoses per 10 high power field [HPF]) in the presence or absence of necrosis were designated as Grade III anaplastic PXAs (Figs 1E and 2E). Immunohistochemically, the tumor cells were positive for glial fibrillary acid proteins and S-100 proteins.

V-raf murine sarcoma viral oncogenes homolog B1 (BRAF)^V600E^ status was evaluated in 29 patients, of which 9 cases were Grade II PXA and 20 cases were Grade III anaplastic PXAs. We found that BRAF^V600E^ mutation was observed in 12 patients (Fig. 1F) and non-V600E mutation in 17 patients (Fig. 2F). We also found that 4 patients (20%) of anaplastic PXAs demonstrated the mutation. The isocitrate dehydrogenases 1 and 2 (IDH1/2) mutation status were evaluated in 36 patients, and we found that IDH1 was mutated in only 1 patient, and not mutated in other 35 patients (Fig. 4D). IDH2 was not mutated in all 36 patients (Fig. 4E). (Table 3)

**Table 3:** Histopathological and molecular findings.

Patient no.	BRAF^V600E^ Status	IDH1 Status	IDH2 Status	Histopathology
1	Mutated	Non-mutated	Non-mutated	Grade II PXA
2	Mutated	Non-mutated	Non-mutated	Grade II PXA
3	Non-V600E mutated	Non-mutated	Non-mutated	Grade III anaplastic PXA
4	Non-V600E mutated	Non-mutated	Non-mutated	Grade II PXA
5	Mutated	Non-mutated	Non-mutated	Grade II PXA
6	Mutated	Non-mutated	Non-mutated	Grade II PXA
7	Mutated	Non-mutated	Non-mutated	Grade III anaplastic PXA
8	Non-V600E mutated	Non-mutated	Non-mutated	Grade III anaplastic PXA
9	Mutated	Non-mutated	Non-mutated	Grade II PXA
10	Non-V600E mutated	Non-mutated	Non-mutated	Grade III anaplastic PXA
11	Mutated	Non-mutated	Non-mutated	Grade II PXA
12	Mutated	Non-mutated	Non-mutated	Grade III anaplastic PXA
13	Non-V600E mutated	Non-mutated	Non-mutated	Grade III anaplastic PXA
14	Non-V600E mutated	Non-mutated	Non-mutated	Grade III anaplastic PXA
15	Mutated	Non-mutated	Non-mutated	Grade III anaplastic PXA
16	Mutated	Non-mutated	Non-mutated	Grade III anaplastic PXA
17	Non-V600E mutated	Non-mutated	Non-mutated	Grade III anaplastic PXA
18	Non-V600E mutated	Non-mutated	Non-mutated	Grade III anaplastic PXA
19	Non-V600E mutated	Non-mutated	Non-mutated	Grade III anaplastic PXA
20	Non-V600E mutated	Non-mutated	Non-mutated	Grade III anaplastic PXA
21	Mutated	Non-mutated	Non-mutated	Grade II PXA
22	Non-V600E mutated	Non-mutated	Non-mutated	Grade III anaplastic PXA
23	Mutated	Non-mutated	Non-mutated	Grade II PXA
24	Non-V600E mutated	Non-mutated	Non-mutated	Grade III anaplastic PXA
25	Non-V600E mutated	Non-mutated	Non-mutated	Grade III anaplastic PXA
26	Non-V600E mutated	Non-mutated	Non-mutated	Grade III anaplastic PXA
27	Non-V600E mutated	Non-mutated	Non-mutated	Grade III anaplastic PXA
28	Non-V600E mutated	Non-mutated	Non-mutated	Grade III anaplastic PXA
29	Non-V600E mutated	Non-mutated	Non-mutated	Grade III anaplastic PXA
30	N/A	Mutated	Non-mutated	Grade II PXA
31	N/A	Non-mutated	Non-mutated	Grade II PXA
32	N/A	Non-mutated	Non-mutated	Grade III anaplastic PXA
33	N/A	Non-mutated	Non-mutated	Grade III anaplastic PXA
34	N/A	Non-mutated	Non-mutated	Grade II PXA
35	N/A	Non-mutated	Non-mutated	Grade II PXA
36	N/A	Non-mutated	Non-mutated	Grade II PXA
37	N/A	N/A	N/A	Grade II PXA
38	N/A	N/A	N/A	Grade II PXA
39	N/A	N/A	N/A	Grade III anaplastic PXA
40	N/A	N/A	N/A	Grade II PXA
41	N/A	N/A	N/A	Grade II PXA
42	N/A	N/A	N/A	Grade III anaplastic PXA
43	N/A	N/A	N/A	Grade II PXA
44	N/A	N/A	N/A	Grade II PXA
45	N/A	N/A	N/A	Grade II PXA
46	N/A	N/A	N/A	Grade II PXA
47	N/A	N/A	N/A	Grade II PXA
48	N/A	N/A	N/A	Grade II PXA
49	N/A	N/A	N/A	Grade II PXA
50	N/A	N/A	N/A	Grade II PXA

N/A = not available.

### Clinical outcomes

As shown in Table 1, 30 patients, including 17 cases with Grade II PXA and 13 cases with Grade III anaplastic PXA, were successfully followed up (range: 3 months to 4.5 years). Out of the 30 patients, 14 cases had evidence of recurrence or progression (including 2 cases with Grade II PXA and 12 cases with Grade III anaplastic PXA), and one case eventually died. The rates of recurrence or progression were higher in patients diagnosed with Grade III anaplastic PXA (92%) compared to those diagnosed with Grade II PXA (12%), with statistical significance (*P* < 0.001).

## Discussion

In 1979, Kepes *et al*.^10^ described for the first time a series of 12 young patients with a distinctive form of astrocytoma was thought to arise from subpial astrocytes due to the presence of “basal lamina”. The astrocytoma was subsequently named as a PXA or Kepes tumor. The typical histopathological features of PXAs include marked cellular pleomorphism with spindle cells, in addition to multi-nucleated giant cells with bizarre nuclei and lipid-filled xanthomatous cells. On immunohistochemical analysis, all tumors are positive for glial fibrillary acid proteins and S-100 proteins^1,2^. In a later study of immunohistochemistry and electron microscope, Giannini *et al*.^11^ revealed the expression of mixed glioneuronal markers in PXAs. Based on the literature, it has been suggested that PXAs arise from less well-differentiated precursor cells with multipotent cell fate potential. According to the WHO classification of CNS neoplasms in 2007^12^, PXA is a distinctive subtype of astrocytoma and is classified as WHO Grade II. The outcomes in cases of PXAs are relatively favorable, with 5- and 10-year overall survival rates of 75–81% and 67–70%, respectively, after total resection^2,13^. Although PXA is generally considered as an indolent neoplasm, it is associated with a higher frequency of recurrence, malignant transformation, and death when compared with other astrocytic tumors of good prognosis, such as pilocytic astrocytomas, indicating the more aggressive nature of PXAs. Lesions with significant mitotic activity (i.e., five or more mitoses per 10 HPF) regardless of the necrosis are designated as PXAs with anaplastic features, as proposed by Giannini *et al*.^2^. It has been reported that 21% of PXA cases in children and adolescents had anaplastic features or malignant transformation at initial presentation^6^. A study dedicated solely to pediatric PXAs found that 27% of cases had anaplastic features^4^. This entity has now been acknowledged and included in the 2016 update of the WHO Classification of Tumors of the CNS as Grade III anaplastic PXA^14^. In our study, the ratio of anaplastic PXAs was about 48%, which was higher than the ratio of anaplastic PXAs in children and adolescents from previous studies, indicating that adult PXAs may be more likely to have anaplastic features. In reviewing the literature, PXAs with anaplastic features have been shown to predict the risk of recurrence^2,15^. A previous study^6^ has also shown the recurrence rates to be higher in patients diagnosed with anaplastic PXAs compared to those diagnosed with Grade II PXAs. Similar results were found in the present study.

PXAs occur primarily in children and young adults, with no bias between genders^1^. PXAs have also been described in infants and elderly patients, with variable prognoses^1,2,13^. The median age of the adult patients in the present series was 36 years old, with two patients older than 65 years. There was a male predominance in the present series, inconsistent with the literature^1^. The typical clinical presentation in cases of PXAs includes a long history of epilepsy, especially in young patients, most commonly in the second decade of life^16^. However, in our adult sample, headaches, which are the atypical clinical presentation, were more common than seizures.

PXAs tend to be superficial and attached to the meninges, with a predilection for the temporal lobe, followed by the parietal, frontal, and occipital lobes^2,13^. Other rare locations are the cerebellum^17^, spinal cord^18^, hypothalamus^19^, pineal region^20^, sella turcica^21^, and retina^22^. In the present series, 44% of cases were superficial in the cerebral hemisphere and attached to the meninges. In these cases, the tumors may have arisen from subpial astrocytes due to the presence of basal lamina, which is a characteristic feature of these astrocytes. In addition, 9 cases of supratentorial PXAs in the present series were adjacent to and even invasion of the lateral ventricles. There are few reports of such an association in the literature, and its significance needs to be confirmed in future studies. Besides, in the present series, PXAs were also observed to be located in the lateral ventricles, dorsal midbrain, and thalamus.

Although there are some imaging reports of PXAs^3–5^, reports dedicated solely to adult imaging studies are rare, and DWI characteristics of adult PXAs have not been previously described. The present study described the MRI metrics of this kind of rare tumor. Consistent with the histopathological descriptor “pleomorphic”, the imaging features of PXAs in the present study were varied, leading to some difficulties in the preoperative diagnosis. Previous reports^3–5^ indicated that PXAs were typically predominantly cystic, with a contrast-enhancing mural nodule close to the meningeal side. However, in our study, only 28% of PXAs presented as a predominantly cystic mass with a mural nodule; 66% of PXAs appeared as a predominantly solid mass with or without cystic changes, whereas others appeared as a predominantly cystic mass with uneven wall thicknesses and without obvious mural nodules. In all 50 cases, the solid component and mural nodule appeared slightly hypo- or iso-intense on T1WI, iso- or slightly hyper-intense on T2WI, and iso- or slightly hyper-intense on T2 FLAIR. The cystic component appeared homogeneously hypo-intense on T1WI, hyper-intense on T2WI, and slightly hypo-intense on T2 FLAIR. The solid components and mural nodules mainly showed marked contrast enhancement, and the cystic wall showed enhancement or no enhancement. Moreover, our findings are similar to those in previously published reports in terms of the frequency of peritumoral edema, with marked peritumoral edema being found in 60% of PXAs. Hemorrhage in PXAs has been described as an uncommon finding, but it was present in 18% of tumors in our series. And a study of PXAs in childhood also reported 33% of the patients had hemorrhage^4^.

Most MRI findings of PXAs are based on single lesions, while reports of multiple lesions are extremely rare. In 2013, Montano *et al*.^23^ reported a primary anaplastic PXA patient with multiple brain lesions located in the region of the left parietotemporal junction, left thalamus, and left hippocampus. They concluded that in this case the PXA was best classified as a multicentric tumor and proposed that multicentricity should be considered as a marker of an anaplastic phenotype in PXA. However, the findings of the present study do not entirely support this proposal. Multiple brain lesions were present not only in the anaplastic PXAs in the current cohort, but also in the Grade II PXA. Due to the similar radiological features of these lesions in different brain regions, we suggest that the lesions in these three cases should be better classified as either disseminated or multicentric. The Grade II PXA case with multiple lesions was concomitant with a spinal intramedullary lesion at the L2 level, and no further lesions were observed along the rest of the spine, suggesting that the lesion possibly represented intramedullary metastasis, although such metastasis is very rare. In the absence of a biopsy or autopsy examination of the spinal lesion, an entity other than a PXA cannot be excluded. The spinal spread of a secondary PXA with anaplastic features is relatively common, occurring in approximately 1/3 of patients^24^. There are very few reports of spinal tumors in patients with primary PXAs without anaplastic features. However, Grade II PXAs also have the biological characteristic of distant spread^25^. The detection of an unresectable spinal lesion underlines the importance of expanding radiological follow-up in PXA patients to imaging of the spine.

DWI has become an important tool in the preoperative characterization of brain tumors. Humphries *et al*.^26^ found an inverse relationship between lesional cellularity and the ADC, with a higher degree of malignancy associated with more intensive tumor spread, and restriction of the free diffusibility of water molecules determining a low ADC *in vivo*. Moore *et al*.^4^ have reported the DWI characteristics of pediatric PXAs. They found that the mean ADC of the tumors was 0.91 ± 0.22 × 10^−3^ mm^2^/s and the mean ADC ratio was 1.14 ± 0.26. These values were lower than those of pilocytic astrocytomas and supratentorial gangliogliomas. This feature is recognized with a similar frequency in the adult PXAs in the present series. In the 23 tumors where the ADC values could be reliably measured, the mean ADC of the tumors was 0.83 ± 0.17 × 10^−3^ mm^2^/s, and the mean ADC ratio was 1.02 ± 0.22. It is important to recognize that the relatively low ADC values and ADC ratios of PXAs in both children and adults are not unusual, which is unlike other low-grade neoplasms.

Recently, the BRAF^V600E^ mutation has been known as a common finding in certain CNS tumors, most commonly in PXAs (nearly 50–60%)^27,28^. Amongst CNS tumors, the presence of BRAF^V600E^ may be helpful in distinguishing PXAs from diffusely infiltrating gliomas that always lack BRAF^V600E^ mutation. Conversely, IDH1/2 mutations, which are frequently present in diffusely infiltrating gliomas, rarely occur in PXAs^29^. Relatively few examples of adult PXAs have been studied for BRAF and IDH1/2 mutation status. Schindler *et al*.^30^ indicated that there may be age-related differences in the BRAF^V600E^ mutation status, based on the fact that 66% of PXAs (63% adult, 69% pediatric) showed this mutation, and 65% of anaplastic PXAs (38% adult, 100% pediatric) showed this mutation as well. Schmidt *et al*.^31^ also reported that 50% (5/10) adult anaplastic PXAs demonstrated the BRAF^V600E^ mutation. Similar to previous research, 41% of PXAs in our study demonstrated the BRAF^V600E^ mutation, and 20% adult anaplastic PXAs demonstrated this mutation. Whereas, in our current series, all cases except one were negative for IDH1 mutation and all negative for IDH2 mutation. Yan *et al*.^29^ has also reported that one case of PXAs was positive for the IDH1 mutation. Identification of BRAF^V600E^ mutations in PXAs could be helpful in deciding the proper therapy. Indeed, for these patients, the use of targeted therapies, such as vemurafenib, was discussed in previous case reports^8,9^. This novel therapeutic approach must be validated, and may ultimately represent a new opportunity for these patients.

## Conclusions

In summary, this study expands our knowledge on the MRI features, molecular markers, and clinical outcomes of adult PXAs, to some extent different from pediatric PXAs. The adult PXAs appear as predominantly solid masses with single lesions in the overwhelming majority of cases, and multiple lesions in few cases. Relatively lower ADC values and ADC ratios of the solid components may distinguish these tumors from other low-grade neoplasms. Anaplastic features are common at initial presentation, conferring more unfavorable clinical outcomes. The evaluation of BRAF^V600E^ and IDH1/2 mutation status may be helpful in distinguishing PXAs from diffusely infiltrating gliomas, and useful for the therapeutic implications of PXAs.

## Methods

### Selection criteria

The data of 50 patients with pathologically-confirmed PXAs between 2011 and 2017 were retrospectively analyzed. All PXA cases were diagnosed according to the 2016 WHO Classification of CNS Tumors^14^. All pathological diagnoses were reviewed by our neuropathologists. BRAF^V600E^ mutation and IDH1/2 mutations were successfully evaluated in 29 and 36 formalin-fixed, paraffin-embedded (FFPE) blocks, respectively. The clinical information, imaging features, pathological changes, molecular markers, medical records and clinical outcomes of these patients were documented.

### MRI protocol and evaluation

All 50 patients underwent MRI scans before surgery, using a 3 T MRI scanner of a variety of manufactures including Siemens, GE, and Philips. The sequences of the plain scan included axial and sagittal T1WI, axial T2WI, and axial T2 FLAIR sequence. DWI, performed on a 3 T Siemens MRI scanner at the same section position as the axial T1WI using a spin echo single-shot echo-planar sequence with the b values of 0 and 1000 s/mm^2^, was available in 26 patients. The ADC maps were generated with a monoexponential model on a voxel-by-voxel basis for all imaging planes. After the patient received intravenous administration of a standard dose of Gd-DTPA (0.1 mmol/kg; Bayer Healthcare Co., Ltd., Guangzhou, China), axial, sagittal, and coronal T1WI sequences were acquired.

Imaging studies were reviewed by two neuroradiologists with 27 and 9 years of experience in neuroimaging. Tumor size, number, location, hemorrhage, degree of peritumoral edema, imaging pattern, signal, and enhancement characteristics were evaluated on MRI images. All the DWI data were transferred to a Syngo workstation for analysis. The solid components of the tumors were sampled by placing up to three nonoverlapping ROIs on the ADC map, avoiding cystic or necrotic parts. If there were multiple brain lesions, the largest one that reflected diffusion-weighted findings for most PXA lesions was selected as the target lesion to increase the accuracy of the measurement. The mean tumoral ADC value was calculated. Further, three ROIs with the same dimensions were placed on the contralesional thalamus because the thalamus maintains normal signal intensity on DWI, even in marked hydrocephalus cases, to serve as a reliable internal control^32^. Then, to assess the objective difference in the ADC value between the tumor and the contralesional brain tissues, the ADC ratio was calculated as the mean ADC of the tumor divided by the mean ADC of the corresponding contralesional thalamus. During the delineation, the two neuroradiologists were blinded to all the clinical information and delineated the characteristics of the tumors from each patient, independently. And they worked together on MRI images of each patient via a consensus reading.

### BRAF^V600E^ and IDH1/2 mutations analysis

Five 10-µm sections from each selected FFPE block were subjected to genomic DNA extraction. DNA was isolated using E.Z.N.A.® FFPE DNA kit (D3399-02; Omega Bio-tek, US) according to the manufacturer’s instructions. Next, the genomic DNA was amplified by polymerase chain reaction (PCR) and the PCR products were purified. The purified products were sequenced and then analyzed using Chromas Lite software. Compared with the reference sequence, the BRAF^V600E^ mutation and IDH1/2 alterations in the hotspot codons R132H and R172K were obtained.

### Statistical analysis

Statistical analysis were performed using SPSS version 21.0 (SPSS, Inc, USA). Data were described as number (percentage), median (range), and mean ± SD. Comparison of the rates of recurrence or progression between groups was evaluated using the Fisher’s exact probability test. The *P* value <0.05 was considered as statistically significant.
